# Predictors of Mortality and Orotracheal Intubation in Patients with Pulmonary Barotrauma Due to COVID-19: An Italian Multicenter Observational Study during Two Years of the Pandemic

**DOI:** 10.3390/jcm13061707

**Published:** 2024-03-15

**Authors:** Nardi Tetaj, Gennaro De Pascale, Massimo Antonelli, Joel Vargas, Martina Savino, Francesco Pugliese, Francesco Alessandri, Giovanni Giordano, Pierfrancesco Tozzi, Monica Rocco, Anna Maria Biava, Luigi Maggi, Raffaella Pisapia, Francesco Maria Fusco, Giulia Valeria Stazi, Gabriele Garotto, Maria Cristina Marini, Pierluca Piselli, Alessia Beccacece, Andrea Mariano, Maria Letizia Giancola, Stefania Ianniello, Francesco Vaia, Enrico Girardi, Andrea Antinori, Maria Grazia Bocci, Luisa Marchioni, Emanuele Nicastri

**Affiliations:** 1 National Institute for Infectious Diseases IRCCS, Lazzaro Spallanzani, 00149 Rome, Italy; nardi.tetaj@inmi.it (N.T.); giuliavaleria.stazi@inmi.it (G.V.S.); gabriele.garotto@inmi.it (G.G.); mariacristina.marini@inmi.it (M.C.M.); pierluca.piselli@inmi.it (P.P.); alessia.beccacece@inmi.it (A.B.); andrea.mariano@inmi.it (A.M.); mletizia.gianicola@inmi.it (M.L.G.); stefania.ianniello@inmi.it (S.I.); francesco.vaia@inmi.it (F.V.); enrico.girardi@inmi.it (E.G.); andrea.antinori@inmi.it (A.A.); mariagrazia.bocci@inmi.it (M.G.B.); luisa.marchioni@inmi.it (L.M.); emanuele.nicastri@inmi.it (E.N.); 2 Department of Anesthesiology, Intensive Care and Emergency Medicine, Fondazione Policlinico Universitario A. Gemelli IRCCS, 00168 Rome, Italy; massimo.antonelli@policlinicogemelli.it (M.A.); joel.vargas@policlinicogemelli.it (J.V.); martina.savino@me.com (M.S.); 3 Department of General and Specialistic Surgery, ICU Policlinico Umberto I, Sapienza University of Rome, 00161 Rome, Italy; f.pugliese@uniroma1.it (F.P.); francesco.alessandri@uniroma1.it (F.A.); giordano.gj@gmail.com (G.G.); p.tozzi@policlinicoumberto1.it (P.T.); 4 Department of Medical-Surgical Sciences and Translational Medicine, Sapienza University of Rome, Sant’Andrea University Hospital, 00189 Rome, Italy; monica.rocco@uniroma1.it (M.R.); annamariabiava@gmail.com (A.M.B.); luigi.maggi@uniroma1.it (L.M.); 5 P.O. “D. Cotugno”, Azienda Ospedaliera dei Colli, 80131 Naples, Italy; raffaella.pisapia@ospedalideicolli.it (R.P.); francescomaria.fusco@ospedalideicolli.it (F.M.F.)

**Keywords:** pneumothorax, pneumomediastinum, COVID-19

## Abstract

**Introduction:** Coronavirus disease 2019 (COVID-19) is a significant and novel cause of acute respiratory distress syndrome (ARDS). During the COVID-19 pandemic, there has been an increase in the incidence of cases involving pneumothorax and pneumomediastinum. However, the risk factors associated with poor outcomes in these patients remain unclear. **Methods:** This observational study collected clinical and imaging data from COVID-19 patients with PTX and/or PNM across five tertiary hospitals in central Italy between 1 March 2020 and 1 March 2022. This study also calculated the incidence of PTX and PNM and utilized multivariable regression analysis and Kaplan–Meier curve analysis to identify predictor factors for 28-day mortality and 3-day orotracheal intubation after PTX/PNM. This study also considered the impact of the three main variants of concern (VoCs) (alfa, delta, and omicron) circulating during the study period. **Results:** During the study period, a total of 11,938 patients with COVID-19 were admitted. This study found several factors independently associated with a higher risk of death in COVID-19 patients within 28 days of pulmonary barotrauma. These factors included a SOFA score ≥ 4 (OR 3.22, *p* = 0.013), vasopressor/inotropic therapy (OR 11.8, *p* < 0.001), hypercapnia (OR 2.72, *p* = 0.021), PaO_2_/FiO_2_ ratio < 150 mmHg (OR 10.9, *p* < 0.001), and cardiovascular diseases (OR 7.9, *p* < 0.001). This study also found that a SOFA score ≥ 4 (OR 3.10, *p* = 0.015), PCO_2_ > 45 mmHg (OR 6.0, *p* = 0.003), and P/F ratio < 150 mmHg (OR 2.9, *p* < 0.042) were factors independently associated with a higher risk of orotracheal intubation (OTI) within 3 days from PTX/PNM in patients with non-invasive mechanical ventilation. SARS-CoV-2 VoCs were not associated with 28-day mortality or the risk of OTI. The estimated cumulative probability of OTI in patients after pneumothorax was 44.0% on the first day, 67.8% on the second day, and 68.9% on the third day, according to univariable survival analysis. In patients who had pneumomediastinum only, the estimated cumulative probability of OTI was 37.5%, 46.7%, and 57.7% on the first, second, and third days, respectively. The overall incidence of PTX/PNM among hospitalized COVID-19 patients was 1.42%, which increased up to 4.1% in patients receiving invasive mechanical ventilation. **Conclusions:** This study suggests that a high SOFA score (≥4), the need for vasopressor/inotropic therapy, hypercapnia, and PaO_2_/FiO_2_ ratio < 150 mmHg in COVID-19 patients with pulmonary barotrauma are associated with higher rates of intubation, ICU admission, and mortality. Identifying these risk factors early on can help healthcare providers anticipate and manage these patients more effectively and provide timely interventions with appropriate intensive care, ultimately improving their outcomes.

## 1. Introduction

Coronavirus disease 2019 (COVID-19) is an infectious disease caused by SARS-CoV-2, responsible for the ongoing global pandemic [1]. The clinical presentation of COVID-19 is highly variable, ranging from asymptomatic infection to acute respiratory distress syndrome (ARDS) and severe systemic disease, which can result in admission to an intensive care unit (ICU) and high mortality rates [2,3,4].

For COVID-19 patients with persistent hypoxemia that is unresponsive to conventional oxygen therapy (COT), it is recommended to initiate therapy with high-flow nasal cannula oxygen (HFNO) or non-invasive positive-pressure ventilation (NIV) as biphasic positive airway pressure (BiPAP) and continuous positive airway pressure (CPAP) when available. If patients fail to respond to these interventions, orotracheal intubation (OTI) and mechanical ventilation (MV) should be initiated [5,6,7,8].

COVID-19 is a significant and novel cause of ARDS [9]. It is worth noting that during the COVID-19 pandemic, the incidence of cases involving pneumothorax and pneumomediastinum, which are forms of pulmonary barotrauma, has increased, particularly among those with COVID-19-related ARDS [10,11]. Pulmonary barotrauma is a severe life-threatening complication that ICU physicians have faced more frequently in recent years due to the pandemic.

Pneumothorax (PNX) is defined as the presence of air in the pleural cavity, with or without lung collapse, while pneumomediastinum (PNM) is characterized by the presence of extraluminal gas within the mediastinum. Subcutaneous emphysema (SE) refers to the infiltration of air in the subcutaneous layer of skin [12].

The literature has reported an increase in the incidence of pulmonary barotrauma (PTX/PNM) as a complication of COVID-19 pneumonia, in the range of 0.05–2% among hospitalized COVID-19 patients [13,14,15,16,17,18,19,20,21], 4–10% among ARDS COVID-19 patients [11,22,23,24,25], and approximately 6–24% among patients requiring mechanical ventilation [26,27,28,29,30,31,32]. The condition is associated with a case mortality rate between 36 and 66%, with a median of 54% (IQR 48–58%) (Table S1, Supplementary Material).

Scientific evidence regarding the factors associated with poor outcomes in COVID-19 patients with pulmonary barotrauma is still lacking.

Therefore, to gain more insight into this subject, we have established the following aims for this multicenter observational study:

- To describe the incidence and the clinical characteristics of COVID-19 patients with pneumothorax and/or pneumomediastinum.
- To identify the factors associated with the risk of death within 28 days of developing pneumothorax and/or pneumomediastinum in COVID-19 patients.
- To identify the factors associated with the risk of OTI after barotrauma in patients in non-invasive mechanical ventilation, as well as the probability of OTI within the first 3 days from the event.

## 2. Materials and Methods

### 2.1. Study Design and Participants

We conducted a multicenter observational study of cases of COVID-19 with PTX and/or PNM from 5 tertiary hospitals serving central Italy between 1 March 2020 and 1 March 2022, including National Institute of Infection Diseases Lazzaro Spallanzani, Rome; Catholic University of Sacred Heart—A. Gemelli Hospital, Rome; Policlinico Umberto I—Sapienza University of Rome, Rome; Sant’Andrea University Hospital, Rome; and V. Monaldi Hospital, Naples.

We included in the study adult patients who had confirmed COVID-19 through a positive nasal/oral pharyngeal or tracheal aspirate swab for reverse transcriptase polymerase chain reaction (rt-PCR) assay, were hospitalized in any of 5 hospitals, and had been diagnosed with pneumothorax and/or pneumomediastinum by at least one imaging modality (including chest X-ray or chest CT scan).

We excluded patients with indeterminate or negative rt-PCR results for SARS-CoV-2 or with no clear evidence of pulmonary barotrauma on radiological imaging, as well as patients with missing data. We also excluded patients who developed iatrogenic pneumothorax (e.g., from central venous catheter insertion, nasal gastric tube, thoracentesis). We collected cases using institutional databases and followed up for at least 28 days after pulmonary barotrauma. We reviewed imaging for all patients throughout their admission. To simplify the reading of the article, we used the common term “pulmonary barotrauma” to refer to the manifestations of the two pathological states, namely PTX and/or PNM.

All clinical and patient management decisions were made by attending physicians according to recommendations from published guidelines and good medical practice [33,34,35]. Patients presenting with dyspnea, hypoxemia, and/or decreasing PaO_2_/FiO_2_ (ratio of arterial partial pressure, PaO_2_, to fractional inspired oxygen, FiO_2_) were initially treated with conventional oxygen therapy (COT), such as low-flow nasal cannula oxygen, simple oxygen mask, and Venturi mask. As the need for oxygen support increased, high-flow nasal cannula oxygen (HFNO) was gradually introduced if available, or non-invasive ventilation (NIV), including continuous positive airway pressure (CPAP) or bilevel positive airway pressure (BiPAP), was introduced according to the patient’s condition. If the patient remained unresponsive to these treatments, they underwent OTI and IMV.

To facilitate the comparison of their clinical characteristics, we categorized all patients into two groups depending on the manifestation of pulmonary barotrauma: those with pneumothorax (with or without pneumomediastinum) and those with pneumomediastinum alone.

We considered the dates 25 June 2021 and 25 December 2021 as the points of demarcation between the periods in which the alpha, delta, and omicron SARS-CoV-2 variants of concern (VoCs) were prevalent in Italy [36].

### 2.2. Data Collection

The data collected from medical records included several factors, such as age, gender, body mass index (BMI), sequential organ failure assessment score (SOFA) within the first 24 h from PTX/PNM, type of supplemental oxygen therapy or ventilation support (COT, HFNO, CPAP, BiPAP and IMV), and the duration until the occurrence of PTX/PNM. Additionally, the diagnosis of PTX/PNM by radiological imaging, hospital length of stay (LOS), chest drainage insertion, presence of subcutaneous emphysema, and outcome (death or discharge) at 28 days from PTX/PNM were also collected.

Therefore, we collected data on the requirement for vasopressor and/or inotropic therapy, including norepinephrine, vasopressin, or dobutamine, used in line with good medical practice [37], administered continuously via intravenous infusion for at least twelve hours within the initial three days after barotrauma.

In addition, we recorded respiratory parameters within the first 24 h from the occurrence of PTX/PNM, which included PCO_2_ (partial pressure of carbon dioxide within arterial blood) and the PaO_2_/FiO_2_ ratio, both measured by arterial blood gas test, as well as PEEP (positive end-expiratory pressure), FiO_2_ (fractional inspired oxygen during respiratory support), and SpO_2_ (saturation of peripheral oxygen).

We recorded the presence of comorbidities among patients, with the most frequent ones being arterial hypertension, cardiovascular diseases, diabetes, obesity (defined as having a BMI > 30 kg/m^2^), chronic pulmonary diseases (which included chronic obstructive pulmonary disease, bronchial asthma, emphysema, or other pulmonary diseases), chronic kidney disease (stage 3–5 of CKD classification by GFR [38], and neoplasm diagnosed within the last 5 years (which includes solid neoplasia or hematological malignancy). These comorbidities were collected as dichotomous variables (yes/no).

All data were collected using a standardized protocol to minimize the possibility of recorder bias. The Institutional Review Board of the respective institutions approved this study, and all data were anonymized to protect the patients’ confidentiality in accordance with the Declaration of Helsinki provisions.

The Strengthening the Reporting of Observational Studies in Epidemiology (STROBE) guidelines were followed when reporting this study.

### 2.3. Statistical Analysis

Quantitative variables were expressed as medians and interquartile range (IQR), while categorical variables were expressed as counts (N) and percentages (%).

The statistical comparison between groups was performed using the Mann–Whitney test for quantitative variables and the Chi-Square test (or Fisher test when necessary) for categorical variables. The Kruskal–Wallis test was used to compare non-normally distributed numerical variables. Statistical significance was defined as *p* < 0.05.

The study initially focused on comparing the clinical characteristics of patients who were categorized based on their diagnosis of pneumothorax with or without pneumomediastinum (named in the study as PTX) and those who had pneumomediastinum only (PNM only). The patients were divided into these different categories to allow a more detailed analysis of pulmonary barotrauma.

We converted some variables into dichotomous variables to perform logistic regression based on established clinical thresholds or value commonly used in several studies and good medical practice. These variables included age ≥ 65 years old (as the median age of our cohort), SOFA score ≥ 4 (as a threshold for clinical severity [39]), PCO_2_ > 45 mmHg (indicating hypercapnia), P/F ratio < 150 mmHg (value used in several studies as a threshold for severe respiratory failure [33]), and PEEP ≥ 12 cmH_2_O (considered a high value in some studies [40]).

Factors associated with the risk of death within 28 days from PTX/PNM diagnosis were identified through logistic regression analysis, with the odds ratios (ORs) and 95% confidence intervals (95% CIs) being reported in univariable analysis, and subsequently with multivariable logistic regression analysis selecting all potential confounding factors through backward elimination, removing from the model all nonsignificant confounders (with *p*-value > 0.10). The adjusted odds ratio (aOR) values and 95% CI were reported. In addition, Kaplan–Meier survival curves were used to represent the statistically significant variables.

Additionally, the probability of orotracheal intubation within three days from the diagnosis of PTX/PNM was assessed using Kaplan–Meier curves.

All statistical analyses were performed using the statistical software SPSS version 27 (IBM Corp., Armonk, NY, USA).

## 3. Results

### 3.1. Clinical Characteristics

**Figure 1:**
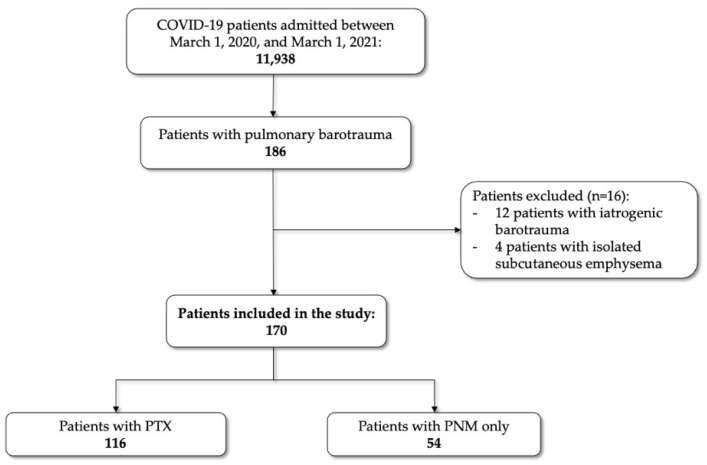
Flowchart showing study selection. PTX, pneumothorax with or without pneumomediastinum; PNM, pneumomediastinum only.

Between 1 March 2020 and 1 March 2022, a total of 11,938 patients with COVID-19 were admitted to the five general hospitals, 1783 (14.9%) of them requiring IMV. Of all admitted patients, 184 patients developed pulmonary barotrauma during hospitalization, but 12 iatrogenic cases and 4 patients with isolated subcutaneous emphysema were excluded from the study analysis. Ultimately, 170 patients with barotrauma met the inclusion and exclusion criteria for the study. Of those, 116 patients had PTX with or without PNM and 54 PNM only, as shown in Figure 1.

The overall incidence of PTX/PNM among hospitalized COVID-19 patients was 1.42%. Specifically, the incidence of PTX with or without PNM was 0.97%, and the incidence of PNM only was 0.45%.

During invasive ventilation, the incidence of PTX with or without PNM was 3.2% (57 cases out of 1783 patients), while the incidence of PNM only in these patients was 0.5% (9 cases out of 1783).

Details on the comparison between the two groups (PTX vs. PNM only) are shown in Table 1, including the fact that 78.4% of PTX cases required chest drainage insertion, while one case with PNM only required chest drainage (1.8%).

**Figure 2:**
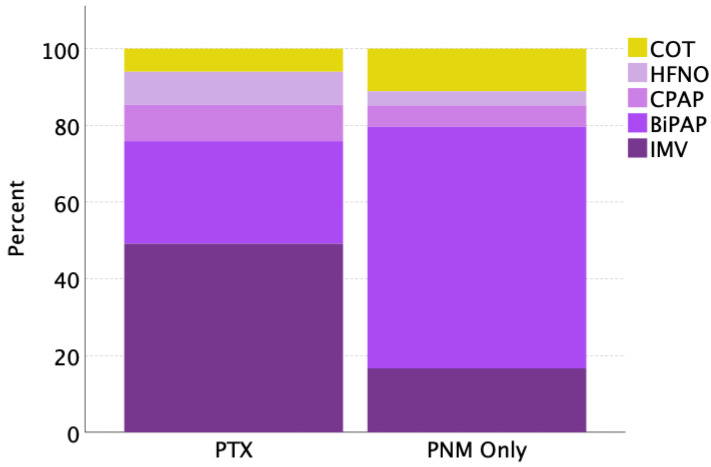
Stacked bar showing the percentages of cases of pneumothorax with or without pneumomediastinum (PTX) and pneumomediastinum only (PNM) categorized by respiratory support. COT, conventional oxygen therapy; HFNO, high-flow nasal cannula oxygen; CPAP, non-invasive continuous positive airway pressure; BiPAP, non-invasive bilevel positive airway pressure; IMV, invasive mechanical ventilation.

Finally, Figure 2 shows the percentages of cases of PTX and PNM only categorized by respiratory support provided in the previous 48 h (including COT, HFNO, CPAP, BiPAP, and OTI). Notably, IMV patients were found to develop PTX more frequently than PNM only (*p* < 0.001), while patients during NIV, specifically during BiPAP, were found to more frequently develop PNM only (*p* < 0.001) (Table 1).

### 3.2. Predictors of Mortality in COVID-19 Patients with Pulmonary Barotrauma

In the univariable analysis, the variables significantly associated with 28-day mortality from pulmonary barotrauma in COVID-19 patients were as follows: SOFA score ≥ 4 within 24 h after the barotrauma (OR 4.89, *p* < 0.001); presence of pneumothorax with or without PNM (vs. PNM only) (OR 2.56, *p* = 0.007) versus pneumomediastinum only; requirement of vasopressor/inotropic therapy after the pulmonary barotrauma (OR 6.83, 95% CI 3.21–14.53, *p* < 0.001); hypercapnia (PCO_2_ > 45 mmHg) and PaO_2_/FiO_2_ ratio < 150 mmHg within 24 h after PTX/PNM (OR 4.84 (*p* < 0.001) and 4.71 (*p* < 0.001), respectively). Additionally, cardiovascular disease was associated with a higher risk of death (OR 5.67, *p* < 0.001). Differently, there was no significant association for age ≥ 65, other comorbidities, PEEP, and different VoCs (Table 2).

The multiple regression analysis shows that several factors were independently associated with a higher risk of death in COVID-19 patients at 28 days of pulmonary barotrauma. These factors include a SOFA score ≥ 4 (OR 3.22, *p* = 0.013), vasopressor/inotropic therapy (OR 11.8, *p* < 0.001), hypercapnia (OR 2.72, *p* = 0.021), PaO_2_/FiO_2_ ratio < 150 mmHg (OR 10.9, *p* < 0.001), and cardiovascular diseases (OR 7.9, *p* < 0.001) (Table 2).

**Figure 3:**
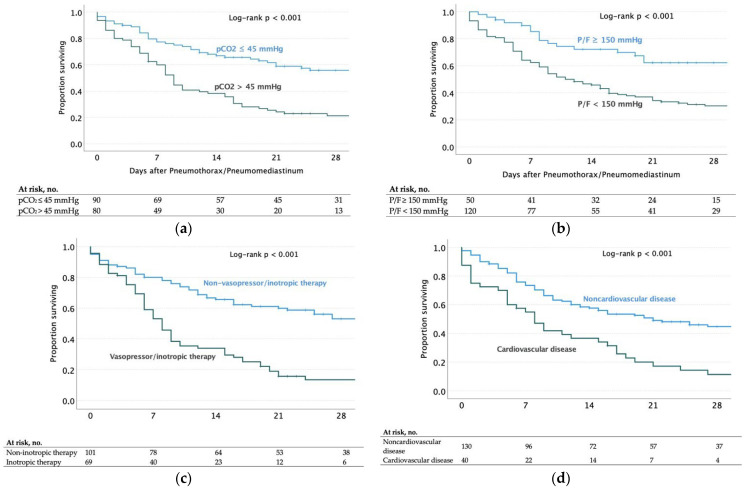
Kaplan–Meier survival curves within 28 days from pulmonary barotrauma, with log-rank *p*, stratified by (**a**) hypercapnia or no hypercapnia (PCO_2_, arterial blood partial pressure of carbon dioxide, > or ≤ 45 mmHg) within 24 h after barotrauma; (**b**) P/F ratio, the ratio of arterial partial pressure to fractional inspired oxygen (< or ≥ 150 mmHg), within 24 h after barotrauma; (**c**) the presence or absence of cardiovascular diseases; (**d**) the requirement for vasopressor/inotropic therapy in continuous intravenous infusion for at least six hours within the first three days after the onset of barotrauma.

Furthermore, Kaplan–Meier survival curves at 28 days after PTX/PNM are represented in Figure 3, highlighting the significant variables.

### 3.3. Predictors and Probability of OTI within 3 Days from Barotrauma Occurring during Non-Mechanical Ventilation

In the study cohort, all deceased patients previously underwent OTI, which allowed for the probability of OTI to be elaborated using logistic regression and Kaplan–Meier curves without any interactions with the event of death, as the event occurred in all cases.

Among COVID-19 inpatients with pulmonary barotrauma that occurred during non-IMV, 64 (61.1%) underwent OTI within three days after barotrauma.

In univariable analysis, as shown in Table 3, we found that the following variables were significant positive predictors for the risk of OTI: SOFA score ≥ 4 (OR 3.6, *p* < 0.004); the requirement of vasopressor/inotropic therapy (OR 3.91, *p* = 0.031); PCO_2_ > 45 mmHg (OR 6.6, *p* < 0.001); and P/F ratio < 150 mmHg (OR 3.4, *p* < 0.007). Cardiovascular diseases in the multivariable analysis were independently associated with a 30% increase in the risk of OTI (OR 1.32, *p* < 0.001). SARS-CoV-2 VoCs were not associated with the risk of OTI.

In addition, after adjusting for potential confounding factors, multivariable logistic regression showed that a SOFA score ≥ 4 (OR 3.10, *p* = 0.015), PCO_2_ > 45 mmHg (OR 6.0, *p* = 0.003), and P/F ratio < 150 mmHg (OR 2.9, *p* < 0.042) were all significant independent factors associated with a higher risk of OTI within 3 days from barotrauma.

**Figure 4:**
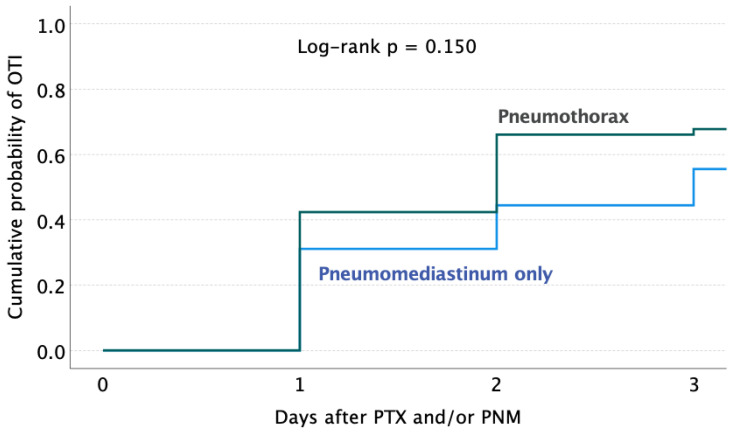
Kaplan–Meier curves estimating the cumulative probability of orotracheal intubation (OTI) at 3 days from the occurrence of pneumothorax (with or without pneumomediastinum) and pneumomediastinum only.

The estimated cumulative probability of OTI in patients after any barotrauma event was 44.0% on the first day, 67.8% on the second day, and 68.9% on the third day, according to univariable survival analysis. In patients who had PNM only, the estimated cumulative probability of OTI was 37.5%, 46.7%, and 57.7% on the first, second, and third days, respectively (see Figure 4).

## 4. Discussion

### 4.1. Clinical Characteristics

This study is one of the largest epidemiological investigations of COVID-19 patients with pulmonary barotrauma, including pneumothorax (PTX) and/or pneumomediastinum (PNM), conducted to date.

The development of pneumothorax during coronavirus infection may be a potentially life-threatening event and negative prognostic indicator [26,41]. However, in our case series, the overall mortality was 58.2% at 28 days from the event, which is consistent with findings from previously published studies (Table S1).

The overall incidence of PTX and/or PNM among hospitalized COVID-19 patients was 1.42%, with PTX (with or without pnm) occurring in 0.97% of cases and PNM only in 0.5% of cases. These rates are in line with previous estimates published so far (Table S1 Supplemental Materials).

The incidence of PTX in mechanically ventilated COVID-19 patients was 3.2%, which is lower than reported rates in previous studies but higher than rates in non-COVID-19 patients despite the widespread use of lung-protective strategies [27,42].

Treatment of PTX was conservative in only 21.6% of cases, with 78.4% of patients requiring chest drainage. Conversely, treatment for PNM was conservative in almost all cases.

Pneumomediastinum-only cases occurred more frequently during non-invasive BiPAP ventilation, while pneumothorax cases were more frequent during invasive mechanical ventilation (Table 1).

One possible explanation for why COVID-19 patients may be more vulnerable to developing pulmonary barotrauma is due to the damage caused by the virus-induced frailty of lung parenchyma. This frailty is thought to result from diffuse alveolar damage, caused by interstitial and alveolar lymphocytic infiltration as well as microvascular thrombosis. These changes have been observed in histologic examinations of affected lungs [43,44,45].

Studies suggest that the Macklin may be a possible pathophysiological mechanism behind pulmonary barotrauma. This effect is characterized by the rupture of alveolar spaces, which may be caused by interstitial inflammation or vascular damage. The resulting dissection and air leak can extend along the bronchoalveolar sheath, ultimately leading to pulmonary lacerations [46,47].

Additionally, in non-mechanically ventilated patients, a prolonged increased respiratory effort often is associated with hyperventilation and coughing fits or non-adaptation to the non-invasive CPAP or BiPAP ventilation. In some cases, this could lead to a pathophysiological mechanism known as patient self-inflicted lung injury (P-SILI). P-SILI has been reported in ARDS patients who were treated with non-invasive ventilation, among both non-COVID-19 [48,49] and COVID-19 patients [50]. The mechanism of P-SILI is related to a deep inspiratory swing of the patients with increased negative pleural pressure and increased transpulmonary pressure, which can predispose the patient to pulmonary barotrauma. While we were unable to prove this in our study due to a lack of data on tidal volume and pressure support, it remains an area of potential investigation for future research [51].

### 4.2. Risk of Death

This observational cohort study identified several risk factors for death in adult COVID-19 patients with pulmonary barotrauma.

In our cohort, we observed that patients with SOFA score ≥ 4, on the day of pulmonary barotrauma, were associated independently with higher odds of in-hospital death (OR 3.07) within 28 days of follow-up from the event, which is in conformity with other studies that previously have shown a correlation with mortality [39]. Indeed, the SOFA score is a good diagnostic marker for sepsis and septic shock and reflects the state and degree of multiorgan dysfunction [52].

Vasopressors and inotropic agents are commonly used in the management of hemodynamic instability in intensive care, especially in case of persistent hypoperfusion after adequate fluid resuscitation or in patients with myocardial dysfunction [53]. COVID-19 patients with PTX and/or PNM may be in cardiac shock and require vasopressor/inotropic therapy. Norepinephrine is the first-line choice for vasopressor therapy in this context, along with fluid resuscitation when necessary. Vasopressin may be considered as an alternative when norepinephrine is not effective [37]. Inotropic agents such as dobutamine may be added to enhance cardiac output in cases of decreased ventricular function.

Our study found that patients who required vasopressor/inotropic therapy (such as norepinephrine, vasopressin, and/or dobutamine) after experiencing barotrauma were associated independently with increased mortality when compared to patients who did not require these therapies. This may be due to the fact that the use of vasopressors and inotropic agents was necessary for patients with hemodynamic instability and that their use in high doses can increase the risk of cardiac arrhythmia and myocardial oxygen consumption, leading to worsened outcomes in critically ill patients [54].

In addition, hypercapnia (PCO_2_ > 45 mmHg) and PaO_2_/FiO_2_ ratio < 150 mmHg, within the first 24 h of experiencing barotrauma, were independent predictors of in-hospital mortality at 28 days from the event. In COVID-19 patients under non-invasive mechanical ventilation (IMV), hypoxia and hypercapnia generate an increase in respiratory rate, leading to dyspnea and prolonged physical effort, eliminating more carbon dioxide and maintaining normal blood PCO_2_. However, in some cases, this mechanism is no longer able to compensate the balance [55]. Indeed, hypercapnia was more frequently present in mechanically ventilated patients and those with pneumothorax (Table 1 and Figure 2).

Hypoxia from both pulmonary barotrauma [56] and ARDS [57] causes hypoxic pulmonary vasoconstriction (HPV) in the pulmonary arterial microcirculation, which may induce a loss of the ventilation/perfusion ratio. HPV leads to a redirection of the blood flow to the alveoli with higher oxygen tension and increasing pulmonary vascular resistance [58]. The increase in pulmonary vascular resistance associated with hypercapnia can adversely affect right ventricular function and hemodynamics [59], worsening the outcome in these patients.

### 4.3. Risk of Intubation

Managing pulmonary barotrauma in COVID-19 patients who are not invasively ventilated can represent a clinical challenge.

In our cohort, all deceased patients underwent orotracheal intubation (OTI) before the event of death, and this allowed us to elaborate the probability of OTI using logistic regression and Kaplan–Meier curves without any interactions with the event of death, because the latter occurred after OTI in all cases.

Among hospitalized COVID-19 patients who experienced pulmonary barotrauma (PTX/PNM) during non-invasive mechanical ventilation, 64 (61.1%) underwent orotracheal intubation within 3 days of the PTX and/or PNM diagnosis.

We observed that SOFA score ≥ 4, PCO_2_ > 45 mmHg, and P/F ratio < 150 mmHg within the first 24 h of pulmonary barotrauma were independent predictors of OTI at 3 days from the event.

The estimated cumulative probability of OTI in patients after the event of pneumothorax with or without pneumomediastinum (PTX) was 44.0% on the first day, 67.8% on the second day, and 68.9% on the third day, according to univariable survival analysis. In patients who had pneumomediastinum only, the estimated cumulative probability of OTI was 37.5%, 46.7%, and 57.7% on the first, second, and third days, respectively (see Figure 4).

Awareness of these risk factors might be useful to physicians in better understanding the patient’s clinical condition and prognosis. This knowledge can also help in evaluating the need for orotracheal intubation and/or admission to the intensive care unit.

Finally, SARS-CoV-2 variants of concern that predominated over the study period, namely alfa, delta, and omicron, were not associated with the risk of death or orotracheal intubation.

### 4.4. Limits and Strength

This study has several limitations. First, it is a multicenter observational study that includes five third-level COVID-19 hospitals. While we attempted to create a homogeneous population in our study and control for confounders, as with any observational study, we acknowledge that our efforts may be incomplete. Some data were not collected by the authors, such as hospital-acquired infections, bloodstream infections, hospital-acquired pneumonia, the vaccination status of the patients, and sequencing for SARS-CoV-2. Therefore, the reported risk factors in our study may have been overstated. Second, the study covers the first 2 years of the COVID-19 pandemic when effective antivirals, medical devices, and adequate adherence to standard supportive therapy were lacking. This might have contributed to the poor clinical outcomes in some patients. Third, we cannot completely exclude the possibility that clinicians may have hesitated to use invasive mechanical ventilation in patients at higher risk of dying due to pre-existing medical conditions, ICU overcrowding, and ventilator shortage in the full pandemic emergency. Fourth, the size of pneumothoraxes and pneumomediastinum were not collected because the diagnosis was made only by chest X-rays in some cases. In addition, given the absence of data on tidal volume and pressure support, the P-SILI phenomenon can only be regarded as a hypothetical explanation in our study. Fifth, while pneumothorax shows signs of clinical worsening and can be more easily recognizable and confirmed by investigation with ultrasound and then with radiological images, pneumomediastinum may not exhibit noticeable clinical signs. Therefore, cases of pneumomediastinum may be underreported. However, this study also has some strengths. Multicenter observational studies are the first step in formulating hypotheses of associations that could incentivize the design of larger studies. The incidences are in line with the literature data (Table S1, Supplemental Data). In addition, by including all adult patients in the five designated hospitals for COVID-19, we believe that our study is representative of cases diagnosed with pulmonary barotrauma in Italy.

## 5. Conclusions

In summary, this large multicenter cohort of COVID-19 patients found an overall incidence of pulmonary barotrauma of 1.42%, which increased up to 4.1% in patients receiving invasive mechanical ventilation.

Physicians should be aware that in COVID-19 patients with pulmonary barotrauma, careful evaluation of factors associated with a higher risk of death is crucial. Such factors include a high SOFA score (≥4), the need for vasopressor/inotropic therapy, hypercapnia, and PaO_2_/FiO_2_ ratio < 150 mmHg, which have been associated with worse outcomes.

These predictors, associated with increased rates of intubation, ICU admission, and mortality, could be valuable in identifying patients with poor prognosis at an early stage, allowing timely interventions with appropriate intensive care. Additionally, these risk factors remained valid during the predominance of different variants of COVID-19.

Understanding the mechanism of associations between these predictors and poor prognosis in COVID-19 patients with pulmonary barotrauma is necessary for developing preventative interventions.

## Figures and Tables

**Table 1:** The characteristics of COVID-19 patients with pulmonary barotrauma.

	Total	PTX	PNM Only	*p*-Value
Number of patients (%)		116	54	
Age, median (IQR)	65.5 (56–73)	66 (56–73)	64.5 (57–70)	0.421
Male, *n* (%)	130	82 (70.7)	48 (88.9)	0.009
Female, *n* (%)	40	34 (29.3)	6 (11.1)	
BMI, kg/m^2^, median (IQR)	27.0 (24–30)	27.1 (24.1–30)	26.1 (24.5–30.5)	0.799
SOFA score *, median (IQR)	5 (3–8)	6 (3–9)	4 (3–6)	<0.001
SARS-CoV-2 Variant of Concern				
Alfa	101 (59.4)	66 (56.9)	35 (64.8)	0.347
Delta	50 (29.4)	36 (31.0)	14 (25.9)	
Omicron	19 (11.2)	14 (12.1)	5 (9.2)	
Respiratory support				
During conventional oxygen therapy, *n* (%)	13 (7.6)	7 (6.0)	6 (11.1)	<0.001
During HFNO, *n* (%)	12 (7.0)	10 (8.6)	2 (3.7)	
During CPAP, *n* (%)	14 (8.2)	11 (9.4)	3 (5.5)	
During BiPAP, *n* (%)	65 (38.2)	31 (26.7)	34 (62.9)	
During IMV, *n* (%)	66 (38.8)	57 (49.1)	9 (16.7)	
Subcutaneous emphysema, *n* (%)	71 (41.7)	45 (38.7)	26 (48.1)	0.252
Chest drain insertion, *n* (%)	92 (54)	91 (78.4)	1 (1.8)	<0.001
Days of hospitalization until PTX/PNM, median (IQR)	9 (4–19)	10.5 (5–21)	7 (4–11)	0.003
Hospital length of stay, median (IQR)	27 (17–43.7)	26 (14–44.5)	31.5 (22–41)	0.628
Requirement of vasopressor/inotropic therapy ^1^, *n* (%)	69 (40.6)	56 (48.3)	13 (24.0)	0.003
Respiratory parameters *				
PCO_2_, mmHg	44 (37–56)	46 (38–58)	41 (36–50)	0.004
P/F ratio, mmHg	119 (90–156)	111 (85–155)	130 (103–157)	0.405
PEEP, cmH_2_O	10 (8–11)	9 (8–10)	10 (8–12)	0.630
FiO_2_, %	65 (55–90)	70 (54–100)	60 (55–70)	0.010
SpO_2_, %	91 (89–94)	91 (88–94)	91 (90–94)	0.332
Comorbidities, *n* (%)				
Arterial hypertension	98 (57.6)	66 (56.9)	32 (59.2)	0.733
Cardiovascular diseases	40 (23.5)	30 (25.8)	10 (18.5)	0.296
Diabetes	29 (17.0)	26 (22.4)	3 (5.5)	0.006
Obesity ^a^	54 (31.7)	41 (35.)	13 (24.0)	0.143
Chronic pulmonary diseases	39 (22.9)	27 (23.3)	12 (22.2)	0.880
Kidney diseases ^b^	10 (5.8)	9 (7.7)	1 (1.8)	0.129
Neoplasia ^c^	16 (9.4)	11 (9.5)	5 (9.2)	0.963
Outcome within 28 days from PTX/PNM				
Mortality, patients (%)	102 (60)	78 (67.2)	24 (44.4)	0.005

Abbreviations: IQR, interquartile range; PTX, pneumothorax; PNM, pneumomediastinum; BMI, body mass index; SOFA score, sequential organ failure assessment score; * the first 24 h from PTX/PNM; HFNO, high-flow nasal cannula oxygen; CPAP, continuous positive airway pressure; BiPAP, bilevel positive airway pressure; IMV, invasive mechanical ventilation; PCO_2_, arterial blood partial pressure of carbon dioxide; FiO_2_, fraction of inspired oxygen; P/F ratio, the ratio of arterial partial pressure (PaO_2_) to fractional inspired oxygen (FiO_2_); PEEP, positive end-expiratory pressure; SpO_2_, peripheral oxygen saturation; ^1^ after the occurrence of PTX/PNM for at least 6 h; ^a^ obesity is defined as BMI > 30 kg/m^2^; ^b^ stage 3–5 of CKD, chronic kidney disease; ^c^ includes solid neoplasia or hematological malignancy in the last 5 years.

**Table 2:** The factors associated with the mortality in COVID-19 patients within 28 days from pulmonary barotrauma.

	All Patients	Exitus ^a^, No. (%)	Univariable		Multivariable	
			OR (95% CI)	*p*	aOR (95% CI)	*p*
Variables	170	99 (58.2)				
Age ≥ 65 years	102	59 (57.8)	1.45 (0.78–2.69)	0.272		
Male, *n* (%) (versus female)	130	73 (56.1)	0.49 (0.22–1.05)	0.069		
SOFA score * ≥ 4	113	82 (72.6)	4.89 (2.47–9.69)	<0.001	3.22 (1.27–8.17)	0.013
Pneumothorax with or without PNM (versus PNM only)	116	78 (67.2)	2.56 (1.32–4.97)	0.005		
Requirement of vasopressor/inotropic Therapy ^1^	69	58 (84.0)	6.83 (3.21–14.53)	<0.001	11.8 (3.97–35.2)	<0.001
SARS-CoV-2 Variant of Concern						
Alfa	101	61 (60.4)	1			
Delta	50	32 (62)	1.07 (0.53–2.14)	0.849		
Omicron	19	11 (52.6)	0.73 (0.27–1.95)	0.614		
Respiratory parameters *						
PCO_2_ > 45 mmHg	80	63 (78.7)	4.84 (2.45–9.55)	<0.001	2.72 (1.16–6.41)	0.021
P/F ratio < 150 mmHg	120	85 (70.8)	4.71 (2.32–9.54)	<0.001	10.9 (3.76–31.8)	<0.001
Comorbidities, *n* (%)						
Arterial hypertension	98	60 (61.2)	1.12 (0.60–2.09)	0.704		
Cardiovascular diseases	34	40 (85)	5.67 (2.03–13.14)	<0.001	7.92 (2.48–25.2)	<0.001
Diabetes	29	22 (75.8)	2.39 (0.96–5.97)	0.056	1.18 (0.36–3.82)	0.077
Obesity ^b^	54	36 (66.7)	1.51 (0.77–2.97)	0.226		
Chronic pulmonary diseases	39	23 (58.9)	0.94 (0.45–1.95)	0.882		
Chronic renal failure ^c^	10	8 (80)	2.80 (0.57–13.6)	0.319		
Neoplasm ^d^	16	12 (75)	2.13 (0.65–6.91)	0.198		

Abbreviations: PTX, pneumothorax; PNM, pneumomediastinum; SOFA score, sequential organ failure assessment score and APACHE II score, acute physiology and chronic health evaluation; PCO_2_, arterial blood partial pressure of carbon dioxide; FiO_2_, fraction of inspired oxygen; P/F, the ratio of arterial partial pressure (PaO_2_) to fractional inspired oxygen (FiO_2_); * within 24 h after PTX/PNM; ^1^ within 1 week after the occurrence of pulmonary barotrauma ≥ 0.1 mcg/kg/min for ≥ 6 h; ^a^ at 28 days after the pulmonary barotrauma; ^b^ obesity is defined as BMI > 30 kg/m^2^; ^c^ stage 3–5 of CKD, chronic kidney disease; ^d^ includes solid neoplasia or haematological malignancy in the last 5 years. OR, odds ratio; CI, confidence interval.

**Table 3:** The factors associated with the risk of OTI after the pulmonary barotrauma in non-mechanically ventilated COVID-19 patients.

	Non-MV Patients	Orotracheal Intubation ^a^, No. (%)	Univariable		Multivariable	
			OR (95% CI)	*p*	aOR (95% CI)	*p*
Dichotomous variables	104	65 (62.5)				
Age ≥ 65 years	57	39 (68.4)	1.75 (0.78–3.90)	0.170		
Male, *n* (%) (versus female)	83	51 (61.4)	0.79 (0.29–2.18)	0.659		
SOFA score * ≥ 4	52	40 (76.9)	3.60 (1.54–8.37)	0.004	3.10 (1.24–7.73)	0.015
Pneumothorax with or without PNM (versus PNM only)	59	21 (35.6)	1.77 (0.75–4.17)	0.199		
Vasopressor/inotropic therapy ^1^	19	16 (84.2)	3.91 (1.06–14.4)	0.031	1.97 (0.47–8.14)	0.347
SARS-CoV-2 Variant of Concern						
Alfa	61	35 (57.4)	1			
Delta	32	25 (78.1)	1.83 (0.76–4.40)	0.195		
Omicron	11	5 (45.5)	0.62 (0.17–2.25)	0.522		
Respiratory parameters *						
PCO_2_ > 45 mmHg	32	28 (87.5)	6.62 (2.10–20.8)	<0.001	6.01 (1.83–19.7)	0.003
P/F ratio < 150 mmHg	77	54 (70.1)	3.41 (1.37–8.48)	0.007	2.87 (1.03–7.93)	0.042
Comorbidities, *n* (%)						
Arterial hypertension	60	39 (65.0)	1.28 (0.57–2.86)	0.539		
Cardiovascular diseases	27	21 (77.8)	2.62 (0.95–7.23)	0.057	1.32 (0.41–4.20)	<0.001
Diabetes	16	13 (81.2)	2.39 (0.96–5.97)	0.056	1.75 (0.41–7.45)	0.639
Obesity ^b^	28	19 (67.8)	1.37 (0.55–3.44)	0.497		
Chronic pulmonary diseases	26	15 (57.7)	0.76 (0.30–1.88)	0.559		
Chronic renal failure ^c^	6	4 (66.7)	1.21 (0.21–6.95)	0.828		
Neoplasm ^d^	13	10 (76.9)	2.18 (0.56–8.47)	0.251		

Abbreviations: Non-MV, non-mechanically ventilated; PTX, pneumothorax; PNM, pneumomediastinum; ^a^ within 3 days from PTX/PNM; SOFA score, sequential organ failure assessment score and APACHE II score, acute physiology and chronic health evaluation; OTI, orotracheal intubation; PCO_2_, arterial blood partial pressure of carbon dioxide; FiO_2_, fraction of inspired oxygen; P/F, the ratio of arterial partial pressure (PaO_2_) to fractional inspired oxygen (FiO_2_); * within 24 h from PTX/PNM; ^1^ after the occurrence of pulmonary barotrauma. Fisher’s exact test. ^b^ obesity is defined as BMI > 30 kg/m^2^; ^c^ stage 3–5 of CKD, chronic kidney disease; ^d^ includes solid neoplasia or haematological malignancy in the last 5 years.

## Data Availability

The data presented in this study are available on request from the corresponding author. The data are not publicly available because of patient privacy and data protection regulations.

## Supplementary Materials

The following supporting information can be downloaded at https://www.mdpi.com/article/10.3390/jcm13061707/s1, Table S1. Previously published cohort studies with ≥50 cases of PTX/PNM in COVID-19 (until June 2022).

## Abbreviations

COVID-19: coronavirus 2019; ICU, intensive care unit; MV, invasive mechanical ventilation; NIV, non-invasive ventilation; CPAP, continuous positive airway pressure; BiPAP, biphasic positive airway pressure; OTI, orotracheal intubation; ARDS, acute respiratory distress syndrome; IQR, interquartile range; BMI, body mass index; SOFA score, sequential organ failure assessment; PaO2/FiO2 ratio, the ratio of arterial partial pressure (PaO2) to fractional inspired oxygen (FiO2); CKD, chronic kidney disease.
