# Supplementary material for: Predictors of Mortality and Orotracheal Intubation in Patients with Pulmonary Barotrauma Due to COVID-19: An Italian Multicenter Observational Study during Two Years of the Pandemic
Source: J Clin Med. 2024 Mar 15;13(6):1707. doi: 10.3390/jcm13061707 (PMC10971014; doi:10.3390/jcm13061707)
Supplement: Supplementary file 1 [file jcm-13-01707-s001.zip › jcm-2890461-supplementary.pdf]

Article

Predictors of Mortality and Orotracheal Intubation in patients with Pulmonary Barotrauma due to COVID-19: An Italian Multicenter Observational Study During Two Years of the Pandemic

Review of literature

Prior to performing the current study, we conducted a literature search for evidence on the subject. We searched Medline and PubMed for original peer-reviewed cohort studies describing the incidence of pneumothorax and pneumomediastinum in COVID-19 between March 2020 and June 2022. Search terms were “pneumothorax” AND/OR “pneumomediastinum” and “COVID-19”, and “Barotrauma” AND “COVID-19”. Inclusion criteria were only reports published in English with at least 50 cases with a denominator of COVID-19 patients. Our search yielded 25 studies. These are detailed in **Table S1** below.

Table S1. *Previously published cohort studies with ≥50 cases of PTX/PNM in COVID-19 (until June 2022).*

Study	Population	Study Type	Single Or Multicenter	Period Of Time	Barotrauma	No. Of Patients	Mortality Of Pnx/Pnm	Outcomes And Incidence Of Barotrauma In COVID-19 Patients	Other Outcomes
**Geraci et al., 2022** [1]	ARDS COVID-19	Retrospective study	Single	1 month	PTX	1595	58%	PTX 7.4%	In-hospital mortality was significantly higher than for those without pneumothorax
**Tetaj et al., 2021** [2]	ARDS COVID-19 in ICU	Retrospective study	Single	1 year	PTX/PNM	497	47.2%	Overall: 7.2%; NIV: 9%; MV 5.8%	The incidence of PNX/PNM in moderate–severe ARDS COVID-19 patients did not differ significantly between the three waves. The incidence of barotrauma in ICU COVID-19 patients in MV was 2.7 events per 1000 invasive ventilator days
**Hamouri et al., 2021** [3]	ARDS COVID-19 in ICU	Retrospective study	Single	3 months	PTX/PNM	239	90.2%	21.3%	Pulmonary barotrauma patients were significantly younger
**Rajdev et al., 2021** [4]	ARDS COVID-19 in ICU	Retrospective study	Single	6 months	PTX/PNM	353	62.5%	NIV: 4.7%; MV:17.3%	-
**Jones et al., 2020** [5]	ARDS COVID-19 in ICU	Retrospective study	Single	1 month	PTX/PNM	83	62.5%	9.6%%	Compared with the non-barotrauma group, a higher proportion of patients with barotrauma died
**Chopra et al., 2021** [6]	ARDS COVID-19 in ICU	Retrospective study	Multicenter	5 months	PTX	842	63%	Overall: 10%; MV: 13%	Mechanically ventilated patients with PTX had worse respiratory parameters at the time of intubation, static respiratory system compliance, and significantly higher in-hospital mortality compared to those without PTX
**Akram et al., 2022** [7]	ARDS COVID-19 in ICU	Retrospective study	Multicenter	8 months	PTX	1788	53.3%	PTX: 4.2%	Pneumothorax is a potential independent risk factor associated with mortality
**Mcguinness et al., 2020** [8]	ARDS COVID-19 in MV	Retrospective study	Multicenter	1 month	PTX/PNM	601	60%	Overall: 15%; PTX: 9%; PNM 10%	Barotrauma is an independent risk factor for death in COVID-19 and is

									associated with a longer hospital stay
**Edwards et al., 2021** [9]	ARDS COVID-19 in MV	Retrospective study	Single	1 month	PTX/PNM	139	46%	9.4%	-
**Housman et al., 2020** [10]	ARDS COVID-19 in MV	Retrospective study	Multicenter	10 days	PNM	171	N.A.	17%	Conservative management of massive subcutaneous emphysema without pneumothorax in COVID-19 patients is safe and limits viral exposure for healthcare workers
**Lemmers et al., 2020** [11]	ARDS COVID-19 in MV	Retrospective study	Single	2 months	PNM	169	56.5%	Overall incidence 13.6%	Seven-fold increase in PTX/PNM as compared with non-COVID-19 group
**Steinberger et al., 2022** [12]	ARDS COVID-19 in MV	Retrospective study	Single	1 month	PNM	363	60%	Overall: 12%	Barotrauma was associated with increased odds of death
**Taha et al., 2022** [13]	ARDS COVID-19 in MV	Retrospective study	Single	1 year	PTX/PNM	334	30%	PTX: 10%; PNM: 6%	Mortality was not significantly higher in barotrauma group. Tocilizumab significantly increased the risk of developing PTX. Presence of pneumothorax was associated with prolonged duration of mechanical ventilation and length of hospital stay
**Belletti et al., 2021** [14]	ARDS COVID-19 in MV	Observational Study	Single	2 months	PTX/PNM	116	60.7%	Overall: 24.1%; PTX: 19%; PNM: 11.2; Both 6%	Ninety-five percent of patients with PNX/PMD had the Macklin effect on a baseline computed tomography scan. Pulmonary barotrauma is associated with increased mortality
**Marciniak et al., 2021** [15]	COVID-19	Prospective observational study	Multicenter	1 year	PTX	131679	-	0.91%	Male sex, smoking, chronic pulmonary disease and invasive ventilation were associated with increased risk of pneumothorax. Pneumothorax is associated

									with increased mortality in COVID-19.
**Dwarakanath et al., 2022**[16]	COVID-19	Retrospective study	Single	1 year	PNM	4131	55%	PNM: 0.92%	PNM is associated with increased mortality
**Muhammad et al., 2022** [17]	COVID-19	Retrospective study	Single	1 year	PTX/PNM	3377	52.6%	Overall: 0.56%	Asthma was a significant risk factor in the development of air leak
**Malik et al., 2022** [18]	COVID-19	Retrospective study	Multicenter	16 months	PTX	103858	49%	1.84%	Pneumothorax increased the risk of death
**Mirò et al., 2021** [19]	COVID-19	Retrospective study	Multicenter	2 months	PTX	71709	32.5%	0.056 %	Spontaneous PTX as a form of COVID-19 presentation is more frequent than in the non-COVID-19 population and could be associated with worse outcomes
**Ekanem et al., 2021** [20]	COVID-19	Retrospective study	Multicenter	3 months	PTX	1619	36%	1.4%	-
**Zantah et al., 2020** [21]	COVID-19	Retrospective study	Single	3 months	PTX/PNM	3368	66.6%	0.66%%	Mortality (66.6%) was not directly related to pneumothorax
**Melhorn et al., 2022** [22]	COVID-19	Retrospective study	Multicenter	5 months	PNM	58484	51.7%	PNM: 0.6%	Mechanical ventilation was the most important predictor of mortality in COVID-19 pneumomediastinum at the time of diagnosis and thereafter along with increased age and diabetes mellitus
**Palumbo et al., 2021** [23]	COVID-19, non-MV	Retrospective study	Single	6 months	PTX/PNM	2635	50%	0.53%	Dexamethasone might induce lung frailty and increase the risk of PNX/PMD
**Greenberg et al., 2021** [24]	COVID-19	Retrospective study	Single	8 months	PTX/PNM	1260	52%	Overall: 2%; PTX: 0.8%; PNM: 0.1%; Both: 0.5	-
**Martinelli et al., 2020** [25]	COVID-19 with barotrauma	Retrospective study	Multicenter	-	PTX/PNM	71	PTX: 63%; PNM: 53%	N.A.	Pneumothorax incidence, age >70 years, and acidosis associated with poor prognosis

References

1. Geraci, T.C.; Williams, D.; Chen, S.; Grossi, E.; Chang, S.; Cerfolio, R.J.; Bizekis, C.; Zervos, M. Incidence, Management, and Outcomes of Patients With COVID-19 and Pneumothorax. *Ann Thorac Surg* **2022**, *114*, 401–407, doi:10.1016/j.athoracsur.2021.07.097.
2. Tetaj, N.; Garotto, G.; Albarello, F.; Mastrobattista, A.; Maritti, M.; Stazi, G.V.; Marini, M.C.; Caravella, I.; Macchione, M.; de Angelis, G.; et al. Incidence of Pneumothorax and Pneumomediastinum in 497 COVID-19 Patients with Moderate–Severe ARDS over a Year of the Pandemic: An Observational Study in an Italian Third Level COVID-19 Hospital. *J Clin Med* **2021**, *10*, 5608, doi:10.3390/jcm10235608.
3. Hamouri, S.; Samrah, S.M.; Albawaih, O.; Saleh, Z.; Smadi, M.M.; Alhazymeh, A.; Syaj, S. Pulmonary Barotrauma in COVID-19 Patients: Invasive versus Noninvasive Positive Pressure Ventilation. *Int J Gen Med* **2021**, *Volume 14*, 2017–2032, doi:10.2147/IJGM.S314155.
4. Rajdev, K.; Spanel, A.J.; McMillan, S.; Lahan, S.; Boer, B.; Birge, J.; Thi, M. Pulmonary Barotrauma in COVID-19 Patients With ARDS on Invasive and Non-Invasive Positive Pressure Ventilation. *J Intensive Care Med* **2021**, *36*, 1013–1017, doi:10.1177/08850666211019719.
5. Jones, E.; Gould, A.; Pillay, T.D.; Khorasanee, R.; Sykes, R.; Bazo-Alvarez, J.C.; Cox, C.; Shurovi, B.; Isted, A.; Simpson, T.; et al. Subcutaneous Emphysema, Pneumomediastinum, and Pneumothorax in Critically Ill Patients With Coronavirus Disease 2019: A Retrospective Cohort Study. *Crit Care Explor* **2020**, *2*, e0210, doi:10.1097/CCE.0000000000000210.
6. Chopra, A.; Al-Tarbsheh, A.H.; Shah, N.J.; Yaqoob, H.; Hu, K.; Feustel, P.J.; Ortiz-Pacheco, R.; Patel, K.M.; Oweis, J.; Kozlova, N.; et al. Pneumothorax in Critically Ill Patients with COVID-19 Infection: Incidence, Clinical Characteristics and Outcomes in a Case Control Multicenter Study. *Respir Med* **2021**, *184*, 106464, doi:10.1016/j.rmed.2021.106464.
7. Akram, J.; Yousaf, Z.; Alabbas, Y.; Almoyaaf, M.I.A.; Ibrahim, A.S.S.; Kharma, N. Epidemiological and Outcome Analysis of COVID-19-Associated Pneumothorax: Multicentre Retrospective Critical Care Experience from Qatar. *BMJ Open* **2022**, *12*, e053398, doi:10.1136/bmjopen-2021-053398.
8. McGuinness, G.; Zhan, C.; Rosenberg, N.; Azour, L.; Wickstrom, M.; Mason, D.M.; Thomas, K.M.; Moore, W.H. Increased Incidence of Barotrauma in Patients with COVID-19 on Invasive Mechanical Ventilation. *Radiology* **2020**, *297*, E252–E262, doi:10.1148/radiol.2020202352.
9. Edwards, J.A.; Breitman, I.; Bienstock, J.; Badami, A.; Kovatch, I.; Dresner, L.; Schwartzman, A. Pulmonary Barotrauma in Mechanically Ventilated Coronavirus Disease 2019 Patients: A Case Series. *Annals of Medicine and Surgery* **2021**, *61*, 24–29, doi:10.1016/j.amsu.2020.11.054.
10. Housman, B.; Jacobi, A.; Carollo, A.; Nobel, T.; Eber, C.; Acquah, S.; Powell, C.; Kaufman, A.; Lee, D.-S.; Nicastri, D.; et al. COVID-19 Ventilator Barotrauma Management: Less Is More. *Ann Transl Med* **2020**, *8*, 1575–1575, doi:10.21037/atm-20-3907.
11. Lemmers, D.H.L.; Abu Hilal, M.; Bnà, C.; Prezioso, C.; Cavallo, E.; Nencini, N.; Crisci, S.; Fusina, F.; Natalini, G. Pneumomediastinum and Subcutaneous Emphysema in COVID-19: Barotrauma or Lung Frailty? *ERJ Open Res* **2020**, *6*, 00385–02020, doi:10.1183/23120541.00385-2020.
12. Steinberger, S.; Finkelstein, M.; Pagano, A.; Manna, S.; Toussie, D.; Chung, M.; Bernheim, A.; Concepcion, J.; Gupta, S.; Eber, C.; et al. Barotrauma in COVID 19: Incidence,

Pathophysiology, and Effect on Prognosis. *Clin Imaging* **2022**, *90*, 71–77, doi:10.1016/j.clinimag.2022.06.014.
13. Taha, M.; Elahi, M.; Wahby, K.; Samavati, L. Incidence and Risk Factors of COVID-19 Associated Pneumothorax. *PLoS One* **2022**, *17*, e0271964, doi:10.1371/journal.pone.0271964.
14. Belletti, A.; Landoni, G.; Zangrillo, A. Pneumothorax and Barotrauma in Invasively Ventilated Patients with COVID-19. *Respir Med* **2021**, *187*, 106552, doi:10.1016/j.rmed.2021.106552.
15. Marciniak, S.J.; Farrell, J.; Rostron, A.; Smith, I.; Openshaw, P.J.M.; Baillie, J.K.; Docherty, A.; Semple, M.G. COVID-19 Pneumothorax in the UK: A Prospective Observational Study Using the ISARIC WHO Clinical Characterisation Protocol. *European Respiratory Journal* **2021**, *58*, 2100929, doi:10.1183/13993003.00929-2021.
16. Dwarakanath, A.; Horgan, L.; Jayawardena, M.; Thirumaran, M.; Johnson, O. The Clinical Course of Pneumomediastinum in Patients with SARS-CoV-2 before Invasive Mechanical Ventilation. *Clinical Medicine* **2022**, *22*, 271–275, doi:10.7861/clinmed.2021-0441.
17. Muhammad, A.I.; Mehta, M.; Shaw, M.; Hussain, N.; Joseph, S.; Vancheeswaran, R. Incidence and Clinical Features of Pneumomediastinum and Pneumothorax in COVID-19 Pneumonia. *J Intensive Care Med* **2022**, *37*, 1015–1018, doi:10.1177/08850666221091441.
18. Malik, S.; Kaushik, C.; Heidelman, E.; Polychronopoulou, E.; Kuo, Y.-F.; Sharma, G.; Nishi, S.P.E. Characteristics and Factors Associated With Mortality in Patients With Coronavirus Disease 2019 and Pneumothorax. *Mayo Clin Proc Innov Qual Outcomes* **2022**, *6*, 257–268, doi:10.1016/j.mayocpiqo.2022.04.003.
19. Miró, Ò.; Llorens, P.; Jiménez, S.; Piñera, P.; Burillo-Putze, G.; Martín, A.; Martín-Sánchez, F.J.; García-Lamberetchs, E.J.; Jacob, J.; Alquézar-Arbé, A.; et al. Frequency, Risk Factors, Clinical Characteristics, and Outcomes of Spontaneous Pneumothorax in Patients With Coronavirus Disease 2019. *Chest* **2021**, *159*, 1241–1255, doi:10.1016/j.chest.2020.11.013.
20. Ekanem, E.; Podder, S.; Donthi, N.; Bakhshi, H.; Stodghill, J.; Khandhar, S.; Mahajan, A.; Desai, M. Spontaneous Pneumothorax: An Emerging Complication of COVID-19 Pneumonia. *Heart and Lung* **2021**, *50*, 437–440, doi:10.1016/j.hrtlng.2021.01.020.
21. Zantah, M.; Dominguez Castillo, E.; Townsend, R.; Dikengil, F.; Criner, G.J. Pneumothorax in COVID-19 Disease- Incidence and Clinical Characteristics. *Respir Res* **2020**, *21*, 1–9, doi:10.1186/s12931-020-01504-y.
22. Melhorn, J.; Achaiah, A.; Conway, F.M.; Thompson, E.M.F.; Skyllberg, E.W.; Durrant, J.; Hasan, N.A.; Madani, Y.; Naran, P.; Vijayakumar, B.; et al. Pneumomediastinum in COVID-19: A Phenotype of Severe COVID-19 Pneumonitis? The Results of the United Kingdom (POETIC) Survey. *European Respiratory Journal* **2022**, 2102522, doi:10.1183/13993003.02522-2021.
23. Palumbo, D.; Campochiaro, C.; Belletti, A.; Marinosci, A.; Dagna, L.; Zangrillo, A.; de Cobelli, F. Pneumothorax/Pneumomediastinum in Non-Intubated COVID-19 Patients: Differences between First and Second Italian Pandemic Wave. *Eur J Intern Med* **2021**, *88*, 144–146, doi:10.1016/j.ejim.2021.03.018.
24. Greenberg, D.J.; Nabors, C.; Chandy, D.; Dhand, A. Pneumothorax and Pneumomediastinum in Patients Hospitalized with Coronavirus Disease 2019 (COVID-19). *Heart and Lung* **2021**, *50*, 386–387, doi:10.1016/j.hrtlng.2021.02.006.
25. Martinelli, A.W.; Ingle, T.; Newman, J.; Nadeem, I.; Jackson, K.; Lane, N.D.; Melhorn, J.; Davies, H.E.; Rostron, A.J.; Adeni, A.; et al. COVID-19 and Pneumothorax: A Multicentre Retrospective Case Series. *European Respiratory Journal* **2020**, *56*, doi:10.1183/13993003.02697-2020.
